# Cell proliferation markers at the invasive tumor front of oral squamous cell carcinoma: comparative analysis in relation to clinicopathological parameters of patients

**DOI:** 10.1590/1678-7757-2016-0238

**Published:** 2017

**Authors:** Aurita Veronica, Beovide CORTEGOSO, Natalia Koerich LAUREANO, Alessandra Dutra da SILVA, Chris Krebs DANILEVICZ, Alessandra Sellinger MAGNUSSON, Fernanda VISIOLI, Pantelis Varvaki RADOS

**Affiliations:** 1 Universidad de la República del Uruguay, Facultad de Odontología, Montevideo, Uruguay.; 2 Universidade Federal do Rio Grande do Sul, Faculdade de Odontologia, Departamento de Patologia Oral, Porto Alegre, RS, Brasil.; 3 Universidade Luterana do Brasil, Faculdade de Odontologia, Departamento de Patologia Oral, Canoas, RS, Brasil.

**Keywords:** Cell proliferation, Oral cancer

## Abstract

**Objectives:**

To evaluate the number of AgNORs *per* nucleus and the expression of Ki-67 at the tumor invasion front (TIF) in relation to clinical parameters (TNM), TIF classification and the prognosis of oral squamous cell carcinomas in an Uruguayan population.

**Material and Methods:**

This study was conducted through a retrospective survey from 2000 to 2010 at the National Institute of Cancer Montevideo, Uruguay and included 40 patients. The samples were obtained from the resection of the tumor and the TIF was defined according with Bryne, et al.^5^ (1992). Expression of Ki-67 was assessed by the percentage of positive tumor cells and the AgNOR was recorded as the mean AgNOR (mAgNOR) and the percentage of AgNOR per nucleus (pAgNOR). All analyzes were performed by a blinded and calibrated observer.

**Results:**

No statistically significant association was observed between immunostaining of Ki-67 and AgNOR with the different types of TIF, regional metastasis and patients prognosis, however it was observed an increase in Ki-67 expression associated with worse patient’s clinical staging, although not statistically significant.

**Conclusions:**

Our results suggest that proliferation markers as AgNOR and Ki-67 are not prognostic markers at the tumor invasive front of carcinoma of oral squamous cell.

## Introduction

Oral cancer is a important global health disease with more than 300,000 new cases annually, totalizing over 275,000 cases and 128,000 deaths *per* year and the morbidity and mortality rates have not improved in the past decades^18^. Studies have shown different patterns of oral squamous cells carcinomas in the various regions of the world, but few studies have evaluated this neoplasm in the Uruguayan population^2,9^. In addition, in Uruguay oral squamous cell carcinoma (OSCC) is diagnosed late, which provides poor prognosis and low survival rate for this disease^20^.

The high failure rate of treatment for patients with oral OSCC suggests the need for better prognostic markers to identify aggressive tumors and tumors that do not respond well to current therapy^3^. The prognostic evaluation by the TNM system is limited because it only evaluates clinical parameters and do not take into account the histopathological characteristics, neither the tumor-host interrelationship^5,17,26^.

The histological features of the OSCC can be widely different from one area to another within the same tumor. It is believed that the most useful site for predicting prognosis is the forehead area of tumor invasion front (TIF) as it would presumably reside more aggressive cells. Morphologically, TIF reflects various molecular interactions that are crucial for the progression of cancer: increased angiogenesis, alteration of adhesion molecules, overproduction of enzymes that degrade the extracellular matrix and an increase in the expression of proteins related to cell proliferation^1,4,5^. The analysis of morphological features such as: degree of keratinization, nuclear polymorphism, pattern of invasion and lymphocytic infiltration of the TIF demonstrated prognostic value as a supplement to the TNM^4,5,17^.

Rapidly growing tumors proved to be deeply invasive and with a poorer prognosis compared with slow-growing tumors. Experimental evidence suggests that the degree of cell proliferation in a tumor is an indicator for estimating biological aggressiveness^12,29^. The increased proliferative capacity may be an early indicator of malignant transformation, and, thus, relevant to analyze the tumor prognosis^7,11^.

Proliferative markers such as argyrophilic nucleolar organizer regions (AgNORs) and Ki-67 have been used to evaluate prognostic significance in OSCC^26,29^. The AgNOR technique consists of silver impregnation of proteins associated with the active nucleoli organizer regions (NORs). Ki-67 is a nuclear antibody that recognizes non-quiescent cells. AgNOR and Ki-67 can provide valuable information about cell proliferation velocity in tumors^25^, and the total fraction of proliferating cells, respectively^10,17,29^.

This theme has been studied by other authors, however so far results are contradictory^17,21,22,28^. To our knowledge, there are no studies in the English literature evaluating concomitantly these proliferation markers (Ki-67 and AgNOR) in TIF in order to predict overall prognosis in OSCC as assessed in our study. So the aim of this research is to evaluate cell proliferation, through the analysis of the number of AgNORs per nucleus and the expression of Ki-67, in the tumor invasion front in comparison with clinical parameters (TNM), TIF classification and the evolution of OSCC in an Uruguayan population.

## Material and methods

This study was conducted through a retrospective survey of the period between January 2000 and December 2010, in the Laboratory of Pathology Anatomy Cancer Institute (INCA) (Montevideo, Uruguay) and approved by the local Ethics Committee with approval protocol number decree 379/08, expedient 091900/000274-13. 109 cases of OSCC were found, of whom 40 met all the inclusion criteria: intra-oral tumors (tumors from lip and oropharynx were excluded), complete clinical data (gender, age, location, TNM), pathology data (clinical stage, degree of differentiation), a clearly demonstrated invasive front and paraffin blocks in good condition and with sufficient material. 69 cases were excluded because did not meet all the inclusion criteria or due to extensive areas of necrosis and poor preservation of the histological structures.

All patients underwent surgical treatment followed by radiotherapy and/or chemotherapy. Samples were obtained from the resection of the tumor, and the TIF was defined as the last six layers of cells in contact with a normal range of connective tissue. The gender, age, location data TNM, clinical staging and evolution of patients, were obtained from medical files. Clinical staging was defined as Stage I (T1N0M0); Stage II (T2 N0M0); Stage III (T1,T2,N1 and M0 or T3,N0,N1 and M0); Stage IVa (T1, T2, T3, N2 and M0 or T4a, N0, N1,N2 and M0)^13^.

Serial sections from tissue samples, 3 µm in thickness, were obtained from paraffin-embedded samples. The first was stained with hematoxylin and eosin to confirm and to classify the field of TIF according to Bryne, et al.^5^(1992). For each tumor, the degree of keratinization, nuclear polymorphism, pattern of invasion and lymphoplasmacytic infiltration were graded and given scores between 1 and 4. The scores were summed into a total score with variable differentiation degree. Score was defined as: 4-8 (good); 9-12 (moderate) and 13-16 (poor). The second section was submitted to immunohistochemical method for the detection of Ki-67 and the third was silver-stained for the detection of AgNORs.

### Immunohistochemistry

The samples were dewaxed and processed for antigen retrieval (pressure cooker, at 125°C for 3 minutes). Endogenous peroxidase was blocked by incubation in 3% hydrogen peroxide in methanol. After washing, the sections were incubated with primary antibody Ki-67, concentration 1:25, (monoclonal anti-human, clone MIB-1, DakoCytomation; Glostrup, Denmark). Envision (DakoCytomation; Carpinteria, CA, EUA) and diaminobenzidine tetrahydrochloride (DAB, DakoCytomation; Carpinteria, CA, EUA) were used to detect specific binding. The sections were counterstained with hematoxylin of Harris, dehydrated and mounted. Positive control was obtained according to the manufacturer.

Microscopic images were captured with an Olympus binocular microscope equipped with an Olympus^®^ video camera [QColor 5, Coolet, RTV and a Dell computer (Dimension 5150)]. Image J software (National Institutes of Health, Bethesda, MD, EUA) was used to count Ki-67 positive cells. 1000 cells were quantified to calculate the percentage of positive cells. Cells with a brown nucleus were considered positive, regardless of the intensity of the color.

### AgNOR

The samples were subjected to the AgNOR technique following the protocol described by Ploton, et al.^24^(1986). AgNORs were quantified according to the criteria established by Crocker et al.^8^(1988). The first 100 well-arranged, non-overlapping nucleated cells were captured at 1000x magnification under immersion oil. AgNOR dots *per* nucleus were quantified on the images captured. Mean AgNORs *pe*r nucleus (mAgNOR) in each sample, and the percentage AgNORs *per* nucleus (pAgNOR>1, pAgNOR>2, pAgNOR>3, pAgNOR>4) were calculated according to the methodology proposed by Xie, et al.^30^ (1997).

### Statistical analysis

All quantification of Ki-67 and AgNORs was performed by a single blinded observer, and the grading scores in TIF were performed by two pathologists (Kappa=0.87).

Statistical analysis was performed using Software GraphPad Prims 5 (GraphPad Software,Inc.; La Jolla, CA, USA). According to date distribution, the comparison of Ki-67 immunostaining and AgNORs was performed by ANOVA followed by *post hoc* Tukey test. Statistical significance level was set at 95% (p<0.05).

## Results

The sample was composed of patients with mean age of 63.3 years, ranging from 40 to 91 years. Most of them were men (77.50%), the tongue was the preferred location (50%) and most of the tumors were T2 (42.50%). No lymph node metastasis (N0) was detected in 60% cases. Regarding the evolution of patients, 57.50% of patients died by the tumor. Clinicopathological features of the 40 tumors samples are summarized in Table 1.

**Table 1:** Clinicopathological features of the tumor samples

Variables		n	%
Age	≥60	17	42.5
	<60	23	57.5
Sex	Men	31	77.5
	Woman	9	22.5
Location	Tongue	20	50.0
	Palate	6	15.0
	Buccal mucosa	7	17.5
	Floor of the mouth	2	5.0
	Trigon retromolar	5	12.5
Evolution	Live	17	42.5
	Dead	23	57.5
T	1	0	0.0
	2	17	42.5
	3	15	37.5
	4	8	20.0
N	N0	24	60.0
	N1	8	20.0
	N2	6	15.0
	N2b	1	2.5
	N2c	1	2.5
M	M0	40	100.0
Clinical Staging	I	0	0.0
	II	12	30.0
	III	14	35.0
	IV	14	35.0

T=tumor size; N0=No regional lymph node metastasis; N1=Metastasis in a single ipsilateral lymph node, 3 cm or less in greatest dimension; N2=Metastasis in a single ipsilateral lymph node, more than 3 cm but less than 6 cm in greatest dimension; multiple ipsilateral lymph nodes, none more than 6 cm in greatest dimension, bilateral or contralateral lymph nodes, no more than 6 cm in greatest dimension; N2b=Multiple ipsilateral lymph nodes, none more than 6 cm in greatest dimension; N2c=Metastasis in bilateral or contralateral lymph nodes, no larger than 6 cm in greatest dimension; M0=Without evidence of distant metastasis; Clinical Staging I=T1, N0 and M0; Stage II=T2, N0 and M0; Stage III=T1, T2, N1 and M0 or T3, N0, N1 and M0; Stage IVa=T1, T2, T3, N2 and M0 or T4a, N0, N1, N2 and M0

**Figure 1:**
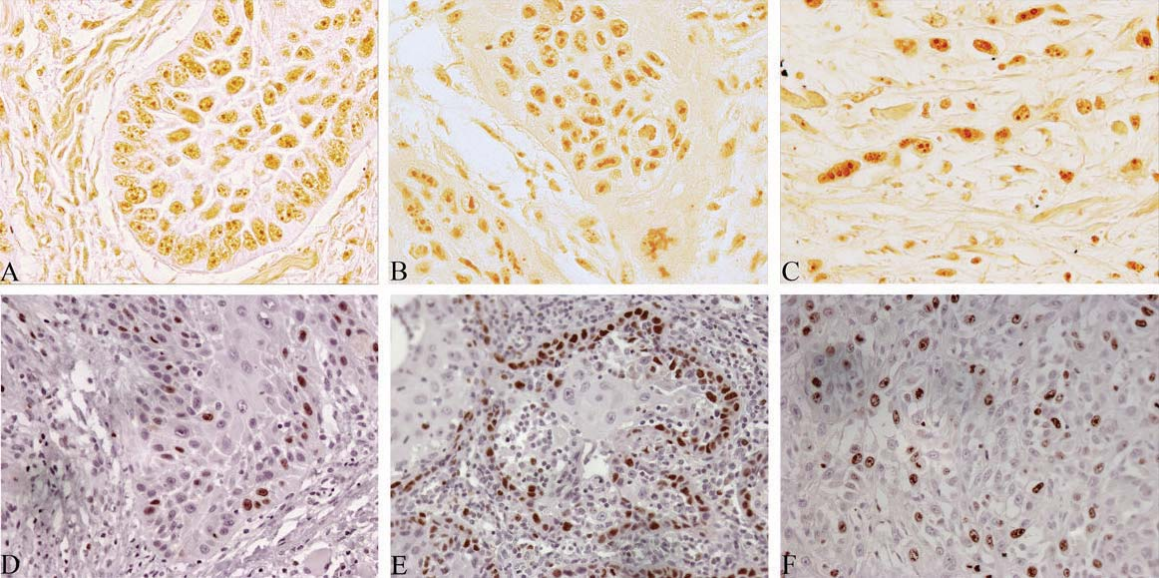
Photomicrography of stained sections of TIF. Good differentiation (A, D), Moderate differentiation (B, E) and poor differentiation (C, F). According to AgNOR staining (A, B, C), 1000x, or Ki-67 immunostaining (D, E, F), 400x

No statistically significant association was observed between immunostaining of Ki-67 and AgNOR with different types of TIF (Figure 1, Table 2). An increase in Ki-67 mean associated with worse patient’s clinical staging was noted, although not statistically significant. However, patients with worse prognosis (died because of the tumor) showed lower mAgNOR (1.85±0.3382) compared to patients with good prognosis (2.07±0.5081), the same was observed regarding Ki-67 quantification (Table 2).

**Table 2:** Proliferative markers, percentage of Ki-67 positive cells and AgNOR, according to Bryne et al.5 (1992) histological malignancy grade of the TIF (tumor invasion front), clinical staging of the tumor, regional metastasis and patient evolution. Legend: mAgNOR=average AgNOR *per* nucleus; pAgNOR=percentage of AgNOR *per* nucleus; Clinical Staging=II - T2, N0 and M0; Clinical Staging=III - T1,T2, N1 and M0 or T3, N0, N1 and M0; Clinical Staging=Iva - T1, T2, T3, N2 and M0 or T4a, N0, N1, N2 and M0; N0=no regional metastasis; N+=presence regional metastasis

Parameters		Mean % Ki-67	mAgNOR	pAgNOR>1	pAgNOR>2	pAgNOR>3	pAgNOR≥4
Classification of TIF [Bryne, et al.^5^ (1992)] Clinical Staging Regional Metastasis	Good	23.30(±14.01)	1.903(±0.2881)	57.64(±13.33)	24.82(±11.72)	7.54(±5.06)	0.18(±0.40)
	Moderate	22.91(±11.71)	1.909(±0.4910)	52.90(±18.11)	24.97(±18.16)	10.73(±13.13)	0.86(±1.69)
	Poor	19.26(±11.54)	1.923(±0.2318)	57.29(±11.28)	27.29(±11.19)	7.14(±3.93)	0.42(±0.78)
	p	0.7826	0.6651	0.5407	0.6131	0.9951	0.6142
	II	19.49(±13.32)	1.869(±0.4277)	55.00(±17.74)	24.75(±16.10)	8.75(±7.65)	0.91(±1.24)
	III	22.43(±11.66)	2.000(±0.3328)	57.63(±12.60)	28.36(±13.73)	11.43(±9.71)	0.92(±2.05)
	Iva	27.20(±14.00)	1.964(±0.5232)	57.36(±16.57)	26.07(±19.74)	10.07(±15.78)	0.35(±1.08)
	p	0.4985	0.5854	0.9049	0.6705	0.5147	0.3300
	N0	23.97(±12.56)	2.023(±0.4705)	58.71(±17.53)	30.29(±18.31)	11.06(±8.62)	0.79(±1.64)
	N+	20.21(±13.31)	1.836(±0.3368)	52.81(±13.39)	20.75(±11.16)	9.47(±13.39)	0.62(±1.36)
	p	0.4205	0.1717	0.2189	0.1032	0.1978	0.7923
Patients Evolution	Live	23.60(±12.85)	2.075(±0.5081)	61.18(±18.04)	32.65(±20.27)	13.12(±15.59)	0.52(±1.73)
	Died	22.23(±12.93)	1.854(±0.3382)	52.78(±13.81)	21.91(±11.20)	7.95(±6.75)	0.86(±1.35)
	p	0.7914	0.1713	0.1186	0.0977	0.4934	0.0917

In relation to regional metastasis, the mean of Ki-67 and AgNOR in the N0 tumor group was higher than in the N+ tumor group, although not statistically significant (Table 2).

## Discussion

The TIF is characterized by cells with a lower degree of differentiation and a higher degree of cell dissociation compared to other parts of the tumor. These cells are more aggressive and tend to invade and metastasize. Recent studies suggest that cells presented at the TIF have different molecular characteristics than those in superficial areas, making the TIF the most important prognostic area of the tumor^1,4,5^. The proliferative activity analysis at the TIF has been studied as an attempt to predict the biological behavior of tumor, as well as the possibility of local recurrence and metastatic potential. However, the proliferative activity at TIF association with clinical pathological parameters remains controversial^16,17,26^.

The cell proliferation markers Ki-67 and AgNOR in TIF of oral cancer were evaluated in a total of 9 studies so far^10,17,19,21-23,26,28,29^. The association with prognosis was evaluated in 5 of these studies, however contradictory results have been observed^21-23,26,29^. In our study it was not observed a clear relationship between prognosis, regional metastasis and cell proliferation at the TIF assessed by immunostaining of Ki -67 and AgNOR counting.

Regarding the clinical evolution, we observed a lower AgNOR counts and Ki-67 positive cells in TIF of patients with worse prognosis, although not statically significant (Table 2). Our findings differ from those of Piffko, et al.^22^ (1997) which observed that OSCC with favorable prognosis contained lower mean AgNOR at the TIF than patients with poor prognosis. This may be due to different methodology, different sample sizes, or a combination of these factors, which could explain the differences between our studies. Piffko, et al.^22^ (1997) performed a morphometric analysis of AgNOR, whereas we performed a quantitative analysis.

In addition, conflicting results in the literature are observed for clinical staging of tumors and proliferative activity of the TIF. Our results demonstrated a tendency of higher mean Ki-67 positive cells in patients with worse clinical staging (stage IV), (not statistically significant). These findings are in agreement with Tumulari, et al.^26^ (2004), which showed that advanced stage clinical (stages III and IV) of the OSCC presented a higher mean of Ki-67 staining when compared the early stage of the disease (stage I and II). However, these results disagree with Piffkó, et al.^23^ (1996), who studied Ki-67 at TIF and Watanabe, et al.^29^ (2010), which noted that the Ki-67 expression was not correlated with clinicopathological features such as survival and clinical staging of OSCC.

Gonzalez-Moles, et al.^15^ (2010), reported that the Ki-67 has no prognostic value in oral cancer, although the authors did not evaluate this marker at the TIF. This can be explained because Ki-67 is a marker of the total fraction of proliferating cells, expressed in all cell cycle phases, excepted G0, being negative to cells in quiescence. Other explanation is that only a small group of cells within the tumor would be responsible for the malignant growth, corresponding to cells in constant proliferation, and the expression of Ki-67 reflects the whole fraction of tumor proliferating cells, what could explain the lack of prognostic value of Ki-67 expression^6,14^.

The results of the present study suggest that the invasive front cells could expend more energy to perform migration and invasion process than to performing cell division. During the tumor invasion, cells undergo epithelial mesenchymal transition, what gives them migratory and invasive properties. The acquired mesenchymal phenotype implies the reorganization of the cytoskeleton what may be incompatible with high cell proliferation^27^. Accordingly with our results, Pereira, et al.^21^ (2016) observed low Ki-67 expression in the TIF and suggests that this may be due to the analysis of cell proliferation has only been performed in TIF, so we can infer that proliferative activity in this region is low and could be influenced by other factors the tumor microenvironment.

Different results between this study and the literature could be explained by differences in criteria for the quantification of Ki-67 and AgNOR, different classification system of TIF evaluated and subjective analysis of immunostaining. Another explanation is that oral cancer may behave differently around the world, since this is the first study of these proliferation markers in an Uruguayan population.

Many aspects may be necessary to establish the real prognosis of OSCC including: clinical staging, the presence of distant metastasis, depth of tumor, location of tumor and perineural invasion^17^. In other words, the establishment of prognosis is complex, since it reflects the characteristics of the entire tumor. Furthermore, the tumors consist of a heterogeneous cell population with biological behavior variable depending on a complex interplay between host and tumor^16,17^.

In conclusion, our results demonstrated that the proliferation at the TIF in OSCC measured by Ki-67 and AgNOR, is not useful to predict patient prognosis. Further studies should be performed in order to clarify whether the tumor invasive front analysis is directly related to prognosis.
